# GagPol-specific CD4^+^ T-cells increase the antibody response to Env by intrastructural help

**DOI:** 10.1186/1742-4690-10-117

**Published:** 2013-10-24

**Authors:** Ghulam Nabi, Michael Storcksdieck Genannt Bonsmann, Matthias Tenbusch, Oliver Gardt, Dan H Barouch, Vladimir Temchura, Klaus Überla

**Affiliations:** 1 Department of Molecular and Medical Virology, Ruhr-University Bochum, Bochum, Germany; 2 Center for Virology and Vaccine Research, Beth Israel Deaconess Medical Center, Boston, USA; 3 Ragon Institute of MGH, MIT and Harvard, Boston, MA, USA

**Keywords:** SIV, HIV VLPs, Gag specific CD4+ T cells, Env antibody response, Intrastructural help

## Abstract

**Background:**

Immunization of rhesus macaques against Gag of SIV resulted in a more rapid appearance of Env antibodies after infection with SIV or SHIV challenge viruses although the vaccines lacked an Env component. We therefore explored whether T helper cells specific for internal HIV proteins could provide intrastructural help for Env-specific B cells and thus increase the Env antibody response.

**Results:**

Mice were immunized by adenoviral vector or DNA vaccines against GagPol and then boosted with virus-like particles (VLP) containing GagPol and Env. Env-specific antibody levels after the VLP booster immunizations were significantly higher in GagPol-immunized mice than in mock-vaccinated controls. Adoptive transfer of CD4+ T cells from GagPol-immunized mice also enhanced the Env antibody response to VLP immunization in the recipient mice. Depending on the presence of VLPs, co-cultivation of CD4+ T cells from GagPol-primed mice with BCR transgenic B cells specific for a protein presented on the surface of the VLPs also resulted in the activation of the B and T cells.

**Conclusions:**

Our study indicates that GagPol-specific T helper cells may provide intrastructural help for Env antibody responses. This cross-talk between immune responses directed against different components of the retroviral particle may be relevant for the immunopathogenesis of retroviral infections and allow to improve virus like particle vaccine approaches against HIV.

## Background

During HIV infection, the immune system encounters the virus in the form of free virions, infected cells, and viral proteins (either soluble or as part of cellular debris). Although the response of the immune system to single HIV proteins has been studied extensively, how immune responses to one viral protein affect the response to a second has been rarely explored. Given the higher-order structures viral proteins are enclosed in, these interactions may be relevant for immune control and immune escape. Suggestive evidence for such a cross-talk of immune responses directed against different lentiviral proteins has been obtained in vaccine studies, in which non-human primates were immunized against Gag of SIV and subsequently challenged with SIV or SIV-HIV hybrid viruses. In at least three independent studies the Env-specific or neutralizing antibody response early after challenge virus infection seemed to be higher in Gag immunized macaques than in the control group [1-3]. In one of these studies, three of four animals immunized by an adenoviral vector encoding Gag had raised neutralizing antibodies at day 40 after SHIV challenge, while none of 4 control animals raised neutralizing antibodies until day 67 after infection [3]. Similarly, Casimiro et al. report weak neutralization titers in Gag-immunized vaccine groups already at day 24 after SIVmac239 infection, while detectable neutralization titers were observed no earlier than day 136 post infection in a subset of control animals. In the third study, macaques with pre-existing anti-Gag responses developed more rapid kinetics of antibody-dependent cell-mediated virus inhibition (ADCVI) which became detectable as early as day 14 after challenge [2].

One explanation by which Gag-specific immunity may increase Env-specific antibody responses is based on intrastructural help, a mechanism described three decades ago for influenza virus and hepatitis B virus [4-8]. Naïve B-cells specific for external viral proteins may take-up the entire viral particle and subsequently present peptides derived from external and internal viral proteins on their MHC-II molecules. Thus, Gag-specific T helper cells induced by vaccination could provide cognate help for Env-specific B cells and thus accelerate the production of Env-specific antibody responses early after infection. To test this hypothesis, we re-analysed data from a non-human primate study, performed immunization and T cell transfer experiments in mice, and explored intrastructural help by *in vitro* B and T cell co-culture experiments.

## Results

**Figure 1:**
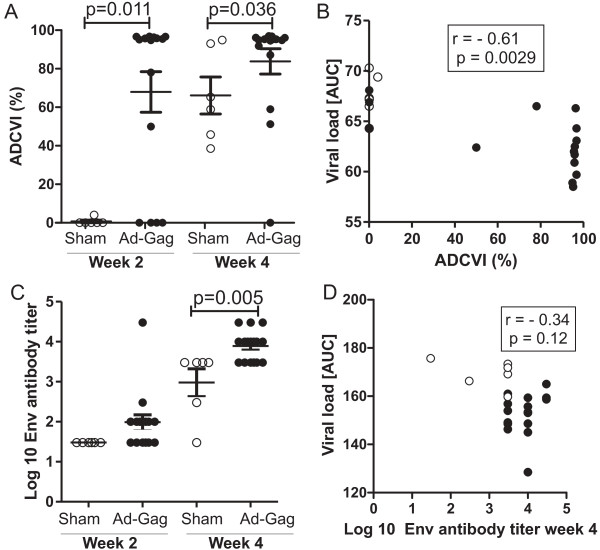
**Antibody responses to Env after SIV infection in macaques immunized against Gag.** ADCVI activity **(A)** and antibody titers to Env **(C)** at 2 and 4 weeks after SIVmac251 infection in control monkeys (sham, n = 6) and monkeys vaccinated against Gag by different prime boost regimens with vectors based on adenovirus types 5, 26, or 35 (Ad-Gag, n = 16). Mean titers with SEM are shown. **B)** Correlation analysis of ADCVI activity and viral load at week 2. **D)** Correlation analysis of Env antibody titers at week 4 with the viral load during the first 4 weeks after challenge. AUC: area under the viral load curve. Groups were compared by the Mann–Whitney test **(A, C)** and data were evaluated by the Spearman rank correlation test **(B, D)**. All data are derived from [2].

To confirm that immunization against Gag enhances the Env antibody response in non-human primates after challenge virus infection we used the comprehensive data set of the study by Liu et al., [2]. In this study, macaques had been immunized with different serotypes of adenoviral vectors encoding SIV Gag either as a homologous or heterologous prime boost regimen inducing a broad spectrum of Gag-specific T cell responses [2]. Depending on the vaccine regimen, peak viral load levels after challenge with SIVmac251 were reduced by 0.5 to 1.4 log. Challenge virus infection resulted in rapid anamnestic Gag-specific cellular and humoral immune responses. Surprisingly, ADCVI activity, which was shown to be dependent on Env-specific antibodies [9], was already detectable in all vaccinated animals two weeks after challenge, while in the mock-vaccinated control animals, ADCVI activity was first observed at four weeks after challenge [2]. The ADCVI activity at two weeks after infection was significantly higher in Gag-immunized macaques than in mock-vaccinated control animals and there was an inverse correlation of ADCVI activity and viral load levels (Figure 1A, B). In line with these findings, the Env antibody titers also followed more rapid kinetics in the vaccinated macaques. Already two weeks after challenge seven of the 16 vaccinated macaques showed an increase in Env specific antibody levels while none of the control animals did. This difference reached statistical significance four weeks after challenge (Figure 1C). However, neither the viral load nor the area under the viral load curve correlated with week 2 or week 4 Env antibody titers (Figure 1D and data not shown).

**Figure 2:**
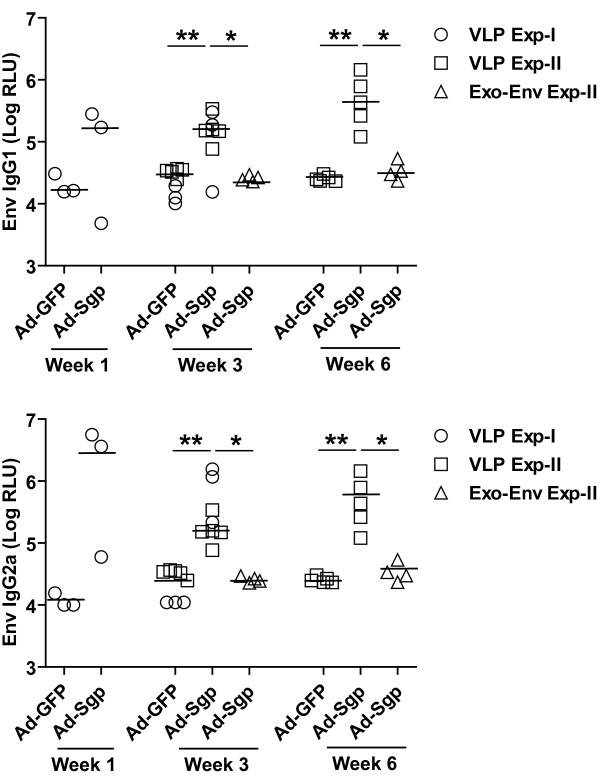
**IgG1 and IgG2a antibody levels to SIVgp130 at 1, 3 and 6 weeks after SIV VLP boost in mice primed 6 weeks earlier with adenoviral vectors encoding SIV GagPol or GFP.** Single and mean values of 3 to 9 animals per group from two independent experiments are given. *P < 0.05, **P < 0.01, Mann Whitney test.

Next, we explored whether T–helper cells specific for Gag or Pol proteins present in the virus particles could directly provide help for Env-specific antibody responses by the intrastructural help mechanism. Mice were primed with an adenoviral vector encoding SIV-Gag and Pol (Ad-Sgp) or an adenoviral vector encoding GFP (Ad-GFP). Six weeks later both groups received virus-like particles (VLP) containing SIV-Gag, Pol, and Env proteins. After the VLP immunization, the Env-specific IgG1 and IgG2a antibody levels were 10 to 50-fold higher in mice primed with the Ad-Sgp vector than in mice which received Ad-GFP (Figure 2). In Ad-Sgp primed mice, the SIV Env-specific antibody response after the VLP immunization was also 10 to 50-fold higher than after booster immunizations with exosomes containing the same amounts of SIV Env as the VLP preparation but lacking GagPol (Figure 2). Since the SIV Env specific antibody response after VLP immunization of mice that had not been primed against SIV GagPol was similar to response after exosome immunization, the enhancement of the Env-specific antibody response in GagPol immunized mice is dependent on the presence of GagPol in the VLPs.

**Figure 3:**
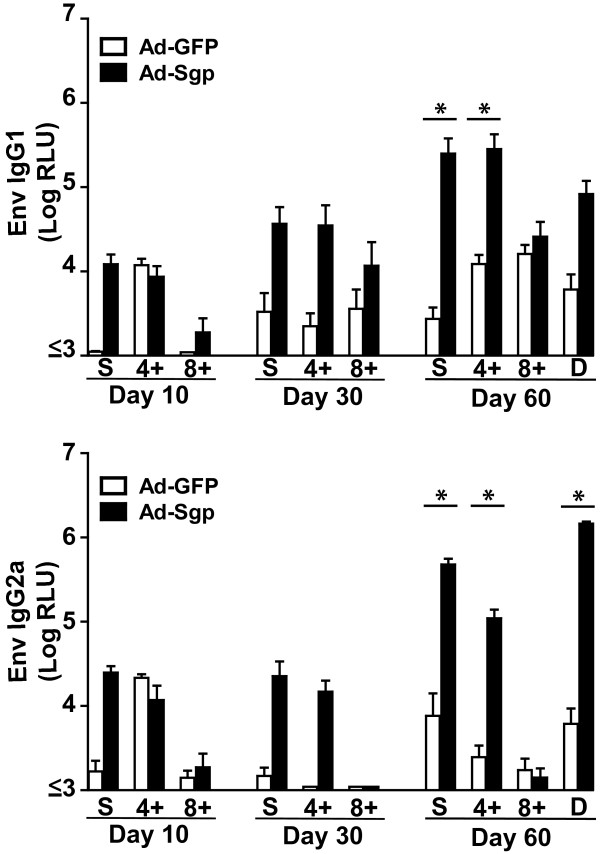
**IgG1 and IgG2a antibody levels to SIVgp130 after cell transfer and subsequent VLP immunization.** Donor mice (D) were immunized with adenoviral vectors encoding SIV GagPol or GFP. Six weeks later splenocytes (S), CD8-depleted splenocytes (4+), and CD4-depleted splenocytes (8+) were transferred into syngeneic recipient mice. The recipient mice and remaining donor mice were immunized with SIV VLPs 5 and 55 days after cell transfer. Mean and standard errors of IgG1 and IgG2a antibody levels to SIVgp130 of four animals per group are shown at the indicated days after the first VLP immunization. Statistically significant differences between the Ad-GFP and the Ad-Sgp group are marked by asterisks (p < 0.05, Mann Whitney test).

To address the question, whether GagPol-specific T-cells induced by prior immunization were responsible for the enhanced antibody response to Env, we performed adoptive transfer experiments. Whole splenocytes, CD4+ T cells or CD8+ T cells of donor mice were isolated six weeks after immunization with either Ad-Sgp or Ad-GFP and transferred into syngeneic recipient mice, which then were immunized with VLPs at day 5 and 55 after transfer. Higher Env-specific IgG1 and IgG2a levels were observed after transfer of either splenocytes or CD4+ T cells from Ad-Sgp immunized donor mice (Figure 3), fully consistent with intrastructural help by Gag and/or Pol-specific CD4+ T cells. Transfer of splenocytes or CD4+ T cells from Ad-GFP immunized mice and CD8+ T cells from both groups of immunized mice did not increase the Env-specific antibody response after VLP immunization in the recipient mice.

**Figure 4:**
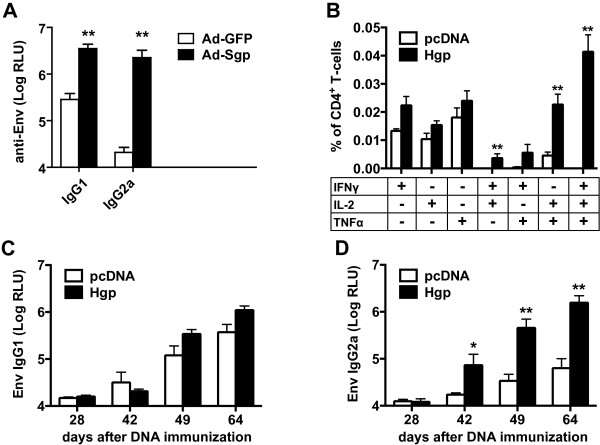
**Intrastructural help for HIV Env. A)** Mice were immunized with Ad-Sgp and boosted with SIV VLPs containing HIV Env 6 and 9 weeks later. Mean and SEM of Env-specific IgG1 and IgG2a antibody levels 2 weeks after the last VLP immunization from 6 animals per group are given. **B-D)** Mice were immunized with an HIV GagPol expression plasmid (Hgp) or empty pcDNA as control by intramuscular DNA electroporation. Gag specific CD4+ T-cell responses were analyzed 14 days after priming by intracellular cytokine staining of in vitro restimulated splenocytes. Shown are the percentages of cytokine producing cells among the total CD4+ T-cell population **(B)**. HIV gp120 specific antibody levels of the subtypes IgG1 **(C)** and IgG2a **(D)** in the sera of immunized mice were determined by ELISA four weeks after DNA prime (day 28), one and two weeks after the first (day 42 and 49) and one week after the second VLP boost (day 64). Columns represent the mean values with SEM of 5–6 animals. *P < 0.05, **P < 0.005, Mann Whitney U test.

Although the exosome experiments described above argue against it, one explanation for the enhanced Env-specific antibody responses could be a cross reaction of SIV Gag or Pol-specific T-helper cells with SIV Env-derived peptides presented on MHC-II molecules. Therefore, Ad-Sgp primed mice were also boosted with VLPs containing SIV-Gag, Pol and HIV-Env. Since HIV and SIV Env only have a 36% amino acid identity a random cross reaction of SIV Gag or Pol-specific T helper cells with a second Env protein seems highly unlikely. Again, HIV-Env specific IgG1 and IgG2a antibody levels were significantly higher in GagPol immunized mice than in Ad-GFP control mice (Figure 4A) further arguing against a cross-reaction on the T helper cell level.

To further extend this observation to HIV particles, mice were also primed against HIV GagPol by a single intramuscular DNA electroporation and boosted five and eight weeks later with VLPs containing HIV Gag, Pol, and Env proteins. The DNA electroporation induced polyfunctional CD4+ T cells specific for immunodominant Gag peptides (Figure 4B). Concomitantly, Env-specific IgG2a levels increased after the VLP immunizations more rapidly and to higher levels in the GagPol-immunized mice than in the control mice (Figure 4D), confirming the observation that GagPol-specific immune responses may increase the antibody response to Env after VLP immunization. A trend to higher Env-specific IgG1 antibody levels was also observed, but this increase did not reach statistical significance and the magnitude of the increase was lower than for IgG2a (Figure 4C).

To corroborate our hypothesis, that GagPol specific CD4+ T cells provide help to B cells specific for surface protein of viral particles we further studied the interaction of B cells and T cells in vitro. Therefore, we took advantage of B cell receptor transgenic mice specific for the model antigen hen egg lysozyme (HEL). The Env protein of HIV VLPs was replaced with HEL by fusing it to the transmembrane and intracytoplasmic domain of the G-protein of vesicular stomatitis virus [10]. To verify that the uptake of VLPs by B cells is BCR dependent we included VLPs lacking HEL in our experiments.

**Figure 5:**
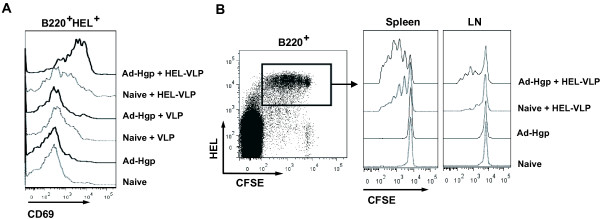
**Activation and proliferation of HEL specific B cells exposed to HEL-VLPs. (A)** Naïve B-cells from SW-HEL mice were co-cultured for one day with splenic DCs and CD4+ T-cells from either non-immunized (naive) or GagPol immunized (Ad-Hgp) mice in the presence of HEL-VLPs or control VLPs lacking HEL. Cells were stained with Alexa488-conjugated HEL and antibodies to B220 and anti-CD69. The CD69 expression pattern of HEL+B220+ cells is shown. The experiment was performed three times with similar results, data of one representative experiment are shown. **(B)** Naïve B-cells from SW-HEL mice were labelled with CFSE and adoptively transferred into non-immunized or GagPol-immunized mice. Two hours later, half of the acceptor mice were injected i.v. with HEL-VLPs. Three days later, spleen and LN cells were stained with Alexa647-conjugated HEL-and anti-B220 antibodies. The CFSE fluorescence intensity of HEL+B220+ cells is shown. Two independent experiments with three to four mice were performed. Representative data are shown.

GagPol-specific T cells were derived from mice immunized against HIV GagPol by adenoviral vector immunization. To avoid potential unspecific stimulation due to the inflammatory effect of adenoviral vector particles, T cells were recovered three to four months after the last adenoviral vector immunization. Co-culture of B-cells from HEL transgenic mice (HEL B cells) with CD4+ T cells from non-immunized mice were used as controls. In the presence of HEL-VLPs and DCs, co-culture of the HEL B cells with the control CD4+ T cells resulted in low level activation of HEL B cells as indicated by upregulation of CD69 (Figure 5A). This activation increased substantially when CD4+ T cells from spleens of GagPol-immunized mice were used and was clearly dependent on the presence of HEL-VLPs (Figure 5A). Co-culture of HEL B cells, with CD4+ T cells from spleens of GagPol-immunized mice and DCs in the presence of VLPs lacking HEL did not increase CD69 expression. Pre-incubation of DCs with HEL-VLPs prior to co-culture with HEL B cells and GagPol-specific T cells did not result in the activation of the B cells either indicating a requirement for direct interaction of HEL-VLPs with the HEL B cells (data not shown). Since the CD4+ T cells were derived from the GagPol immunized mice more than 12 weeks after the last adenoviral vector immunization antigen unspecific activation of the HEL B cells by the CD4+ T cells from the adenoviral vector immunized mice was considered to be highly unlikely.

To check if our in vitro results directly translate into the animal model, we adoptively transferred CFSE-labelled HEL B cells in GagPol immunized or non-immunized mice. The immunized recipient mice received the HEL B cells 13 weeks after the last adenoviral vector immunization to avoid an influence of the inflammatory response induced by adenoviral vector particles. An extensive proliferation of the transferred HEL B cells was observed only in GagPol immunized mice receiving HEL VLPs (Figure 5B), which is fully consistent with a stimulatory effect of GagPol-specific T helper cells on B cells specific for the surface protein of VLPs.

**Figure 6:**
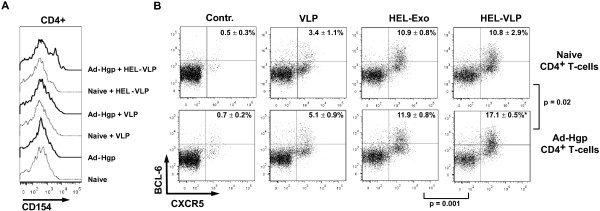
**Activation and differentiation of CD4+ T cells by HEL-specific B-cells exposed to HEL-VLPs.** CD4+ T-cells from non-immunized (naive) or GagPol immunized (Ad-Hgp) mice were co-cultured with naïve B-cells from SW-HEL mice and splenic DCs in the presence of HEL-VLPs or control VLPs lacking HEL. **(A)** After one day, cells were stained for CD4 and the CD-154 activation marker. The experiment was performed three times with similar results, data of one representative experiment are shown. **(B)** After 6 days of incubation, cells were stained for CD4, intracellular BCL-6, and CXCR-5. Follicular-helper cells were defined by co-expression of BCL-6 and CXCR-5. Numbers in upper right corners indicate mean percentage ± standard deviation of follicular-helper cells among total CD4+ T cells from three independent experiments. Each of the three experiments was performed in duplicates and the mean of the duplicates was used for statistical analysis of the mean of the three experiments. P values of unpaired t-tests for the indicated group comparisons are shown.

In addition to the stimulation of B cells, we further analyzed the activation of CD4+ T cells in our in vitro co-cultures. The interaction of CD4+ T cells from GagPol immunized mice, HEL-specific B cells, and DCs in the presence of HEL-VLPs resulted in the upregulation of CD154 (CD40L), indicating that the T cells became competent to provide help for cognate B cells (Figure 6A). CD4+ T cells from non-immunized mice were not activated by co-incubation with DCs, HEL-B cells, and HEL-VLPs. Co-culture of CD4+ T cells from GagPol-immunized mice with DCs, HEL-B cells and VLPs lacking HEL did not result in CD4+ T cell activation either. This indicates that B cells can indeed take up HIV-VLPs by BCRs specific for proteins from the surface of the VLPs and present internal GagPol-derived peptides to GagPol-specific T cells.

To further explore the outcome of the interaction between DCs, B cells, and CD4+ T cells in the presence of VLPs containing matched B and T cell epitopes from different proteins of the VLP, the differentiation of the CD4+ T cells into follicular T helper cells (fTH) was investigated by determining co-expression of CXCR5 and BCL-6 (Figure 6B) as an established indicator of the fTH phenotype. Co-culture of B cells from SW-HEL mice with T cells from either naïve or Ad-Hgp immunized mice did not induce fTH cells (Contr. column in Figure 6B), indicating that CD4+ T cells from Ad-Hgp mice do not lead to a high background by themselves. Adding VLPs lacking HEL to these co-cultures we observed a trend to a higher percentage of fTH cells in the presence of CD4+ T cells from GagPol primed mice (VLP column in Figure 6B). As a source of B-cells SW-HEL BCR-transgenic mice on a RAG +/+ background were used. Therefore, only up to 40% of the B-cells from these SW-HEL mice carry the HEL-specific BCR, while the rest of the B cell population has undergone V_H_ gene replacement leading to a broad BCR repertoire. Therefore, these non-HEL B-cells may also take-up VLPs in a BCR-dependent or independent manner and present HIV GagPol-derived peptides on MHC-II molecules explaining the observed trend to a higher percentage of fTH cells in the presence of CD4+ T cells from GagPol-primed mice. Adding exosomes containing HEL, but lacking GagPol, also enhanced fTH induction. However, this was clearly independent of the Ad-Hgp immunization (HEL-Exo column in Figure 6B) and may be explained by more efficient uptake of the HEL exosomes by the HEL B cells and subsequent presentation of exosome-derived peptides from human proteins to the T helper cells derived from GagPol-primed and naive control mice. In the presence of naïve T cells, addition of HEL-VLPs resulted in a similar percentage of fTH as observed for HEL exosomes. Importantly, co-culture of HEL-VLPs with HEL B cells and CD4+ T cells from GagPol-primed mice induced a significantly higher percentage of fTH cells than the corresponding co-culture with T helper cells from naïve mice. In addition, the percentage of fTH cells in the presence of GagPol primed T cells was significantly higher after stimulation with HEL-VLPs than HEL exosomes demonstrating the specificity for GagPol. The follicular T helper phenotype was further confirmed by GL7 positivity of nearly all of the CD4+ BCL-6 + CXCR-5+ (data not shown). Omitting HEL B cells from the co-culture blocked differentiation of the T helper cells into fTH completely (data not shown). In the absence of DCs, differentiation into fTH by HEL-VLP exposed B cells was detectable, but less efficient. Thus, most efficient induction of fTH cells required triggering of cognate T- and B-cells by their respective antigens.

## Discussion

Our mouse experiments clearly demonstrate that GagPol-specific CD4+ T cells can increase the Env antibody response after exposure to virus-like particles of SIV and HIV. Mechanistically, our results are fully consistent with the intrastructural help hypothesis, by which B-cells specific for external viral proteins take-up the entire viral particle and subsequently present peptides derived from external and internal viral proteins on their MHC-II molecules. Thus T helper cells specific for the peptides from the internal viral protein may provide cognate help for B cells encoding antibodies against the external protein.

Whether the intrastructural help observed in the present study using VLP immunization is similar to that occurring during viral infection is unclear. This may largely depend on the relative contributions of virions, cell-associated viral antigens, or soluble viral proteins on the antibody response to the viral surface protein. Priming of mice with influenza virus cores or purified M protein clearly enhanced the antibody response to HA after influenza virus infection [7,8]. In transfer experiments, HA-, M-, and NP-specific T helper cell clones also enhanced the HA-antibody response to influenza virus infection in nude mice to a similar extent [5]. In contrast, a vaccinia virus infection model revealed that CD4+ T cells specific for an epitope of one protein of the vaccinia virus particle did not enhance the antibody response to another protein of the virion [11]. In addition, there was an extensive overlap of vaccinia virus proteins recognized by T-helper cells and targeted by antibodies indicating that individual proteins are the unit of T cell – B cell interaction at least for a large virus [11].

Whether intrastructural help leads to a modulation of the antiviral immune response during natural HIV/SIV infection is unknown and difficult to explore. Faster kinetics of the antibody titers to Env and the ADCVI activity observed in non-human primates vaccinated against Gag after challenge virus infection provide some evidence. However, secondary effects such as differences in the preservation of immune competence need to be excluded. Although we did not observe an inverse correlation between viral load and Env antibody titers, further analyses on the correlation of Env antibody levels and Gag-specific cellular immune responses early after challenge virus infection are needed to further strengthen the hypothesis.

In contrast to the Env antibody titers as determined by ELISA, the ADCVI activity correlated inversely with viral load suggesting that ADCVI may contribute to control of virus replication. Since the Env antibody titers and the ADCVI activity do not correlate (data not shown), the two parameters of the Env antibody response probably reflect different functionalities of Env antibodies. This suggests that intrastructural help can not only be exploited to increase the Env antibody responses, but also to modulate it by for example preferential induction of antibody isotypes conferring ADCVI activity. Preferential stimulation of HIV Env-specific IgG2a antibody responses in mice immunized against HIV GagPol by DNA electroporation provides some experimental support for such an approach.

Our observation that GagPol-specific T helper cells may provide help for Env antibody responses has implications for the conclusions that can be drawn from T cell based vaccine studies on effector mechanisms mediating control of virus replication. Since Env is the only viral protein of the intact virion accessible to antibodies, durable control of virus replication after immunization with vaccines lacking an Env component has been taken as evidence for the potency of cytotoxic T cells. Due to intrastructural help, the antibody response to the Env of the actual challenge virus may be raised more rapidly and could thus contribute to control of virus replication by neutralization or non-neutralizing antibody-dependent effector mechanisms, like ADCVI. At the same time, intrastructural help by GagPol-specific T helper cells may be an effective way to improve the efficacy of vaccines originally designed to raise protective Env antibody responses. In the absence of sterilizing immunity, break-through virus infections may be controlled more efficiently by a more rapid ADCVI activity facilitated by GagPol-specific T helper cells.

The VLPs used in the present study are not only tools to dissect the interaction of viruses with the host immune system, but are also promising vaccine candidates. Presentation of the Env protein in its native membrane-embedded conformation and increased immunogenicity in comparison to soluble proteins are favourable characteristics of VLP vaccines. Thus, it may also be possible to exploit the intrastructural help effect observed in the present study to enhance and modulate the immune responses raised by VLP vaccine candidates using GagPol-specific T helper cells.

## Conclusions

GagPol-specific T helper cells can clearly increase the Env antibody response to VLP immunization and may also explain the higher Env antibody responses during breakthrough infections in non-human primates only immunized against Gag. It remains to be determined how this cross-talk between immune responses directed against different components of the retroviral particle affects the immunopathogenesis of retroviral infections. Intrastructural help is an attractive strategy to increase and modulate the immunogenicity of VLP vaccines.

## Methods

### Mice

Mice were housed at the animal facility of the faculty of medicine, Ruhr University Bochum, Germany in singly-ventilated cages in accordance with the national law and institutional guidelines and were handled according to the Federation of European Laboratory Animal Science Association. Six to eight weeks-old C57BL/6J mice (BL6), BALB/c and SW-HEL mice were used in this study. The BCR-transgenic SW-HEL mice were kindly provided by Dr. A. Freitas, The Lymphocyte Population Biology Unit, Pasteur Institute, France.

### DNA immunization

The codon-optimized HIV GagPol expression plasmid Hgp (designated Hgpsyn in [12]) and the empty vector pcDNA3.1 (Invitrogen, Carlsbad, CA, USA) were prepared using the NucleoBond® Xtra Maxi EF Kit (Macherey-Nagel, Düren, Germany) and diluted in sterile PBS. The mice were anaesthetized by intraperitoneal injection of 50 mg/kg body weight Ketamin (CP-Pharma, Burgdorf, Germany) and 10 mg/kg body weight Xylazin (Bayer, Leverkusen, Germany). The hind legs were shaved prior to insertion of the TriGrid™ electrode array (Ichor Medical Inc., San Diego, USA) bearing the centred injection needle. Fifty μl containing 10 μg Hgp + 10 μg pcDNA3.1 or 20 μg pcDNA3.1 alone were injected intramuscularly in each hind leg followed by the electric pulse.

### Adenoviral vector immunization

The recombinant adenoviruses Ad-Hgpsyn expressing codon-optimized HIV-GagPol and Ad-Sgpsyn expressing codon-optimized SIV-GagPol have been described previously [13,14] and are designated Ad-Hgp and Ad-Sgp in this manuscript respectively. Ad-GFP expressing GFP has also been described previously [14]. The adenoviral vectors were purified from T-Rex-293 cells (Invitrogen, Karlsruhe, Germany) by CsCl density gradient centrifugation as reported previously [13]. The concentration of the adenoviral vector particles in the vector stocks was determined by measuring the optical density at 260 nm.

For the adenoviral vector prime VLP booster immunization experiments, 6–8 weeks old BALB/c mice were immunized subcutaneously with 1×10^9^ particles of Ad-Sgp, Ad-Hgp or Ad-GFP. For in vitro experiments, BL6 mice were immunized twice with a two month interval with 10^10^ particles of Ad-Hgp. Antibody responses to Gag confirmed the immunogenicity of the vector immunization.

### VLP production and immunization

For efficient incorporation of SIVgp140 and HIVgp140, into VLPs, the respective coding regions were fused in frame to the intracytoplasmic domain of VSV-G. The HEL incorporation into VLPs was facilitated by the HEL coding region fused in frame to the transmembrane and intracytoplasmic domain of VSV-G. The expression plasmid SIVgp140-GTM contains a codon-optimized sequence coding for amino acid 23 to 682 of Env of SIVmac239 (numbering according to Genbank entry M33262.1 fused to amino acid 97 to 122 of VSV-G (Genbank entry CAA24524.1). pConBgp140GCD contains a codon-optimized clade B consensus sequence encoding amino acid 1 to 703 (Genbank entry ABG67916.1) fused to the intracytoplasmic domain of VSV-G (Amino acid 97 to 122, Genbank Entry CAA24524.1). pHEL-GCD encodes amino acid 1 to 147 (numbering according to Genbank entry ACL81762.1) fused to the transmembrane and intracytoplasmic domain of VSV-G (Amino acid 52 to 122 according to Genbank entry CAA24524.1). SIV virus like particles were produced by transient co-transfection of HEK293T cells with Sgpsyn [12] and SIVgp130-GTM by the calcium phosphate coprecipitation method.

SHIV, HIV, and HEL VLPs were prepared by transient transfection of HEK 293T cells with the polyethylenimine method [15]. In brief, one day prior to transfection 293T cells were seeded in 175 cm^2^ cell culture flasks (Greiner Bio One, Frickenhausen, Germany) to reach 60 - 80% confluency on the next day. For SHIV VLPs 35 μg Sgpsyn and 35 μg pConBgp140GCD, for HIV VLPs 35 μg Hgp and 35 μg pConBgp140GCD, and for HEL VLPs 35 μg of Hgp with 35 μg of pHEL-GCD were mixed in 5 ml serum free DMEM (Invitrogen, Carlsbad, CA, USA) per 175 cm^2^ flask. Subsequently, 105 μl of 1 μg/μl polyethylenimine (1:1.5 DNA to polyethylenimine) were added and the solution was thoroughly mixed. Following 15 min incubation at RT the transfection mixture was added to the cells. After 6 h the medium was exchanged with a 1:1 mixture of serum free DMEM and AIM-V (Invitrogen, Carlsbad, CA, USA). To harvest the VLPs the supernatant was collected 48 h after transfection and centrifuged for 10 min at 940× g to remove dead cells and cellular debris. Following filtration through a 0.45 μm filter the VLPs were purified by ultracentrifugation through a 20% sucrose cushion for 2.5 h at 90,000× g at 4°C. After discarding the supernatant the VLPs were resuspended in PBS and stored at -80°C.

The VLPs were characterized by Western Blot using serum from an SIV-infected macaque (serum 1604, German Primate Centre) or HIV-IG (BP1035 - Acris antibodies GmbH). To determine the amount of Env and HEL in the VLP preparations, high binding microtiter plates (Greiner Bio One, Frickenhausen, Germany) were coated with the purified VLPs or serial dilutions of known amounts of SIVgp130 (EVA670, NIBSC), HIVgp120 (HIV-1 IIIB, NIH Reference and Reagent Program) or HEL (Sigma Aldrich). The amounts of SIVgp130, HIVgp120, or HEL from the VLP preparations bound to the microtiter plate were then determined using the monoclonal KK45 SIV gp130 antibody (NIH AIDS Reference and Reagent Program), the monoclonal gp120 antibody 2G12 (Polymun), a rabbit anti-HEL serum (Fitzgerald Industries), and matched HRP-conjugated secondary antibody reagents.

Exosomes containing SIV Env or HEL were prepared and characterized as described for SIV VLPs and HEL VLPs, respectively, by omitting the GagPol expression plasmids from the transfection reaction.

For the mouse immunization studies the SIV VLPs were diluted in sterile PBS to a final concentration of 3 μg/ml gp130 and the HIV VLPs to 4 μg/ml gp120. Mice were immunized by subcutaneous injection of 100 μl distributed into both hind foot pads.

To monitor humoral immune responses mice were bled by puncture of the retro-orbital sinus with a heparinized micro-hematocrit capillary. Sera were obtained by centrifugation for 5 min at 2600× g in a table top centrifuge and stored at -20°C until use.

### Determination of antibody levels

SIV and HIV Env-specific humoral immune responses were analyzed by a gp130 and gp120 specific ELISA respectively. Both proteins were expressed in HEK 293T cells with a C-terminal His-Tag and purified using the ProPur Midi MC kit (Nunc, Denmark) according to the manufacturer’s instructions. The protein preparations were 90–95% pure as judged by Coomassie staining. Briefly, white 96-well highbinding microtiter plates (Greiner Bio One, Frickenhausen, Germany) were coated with 200 ng of SIVmac239 gp130 or 100 ng of HIV-1 consensus clade B gp120 in 0.1 M bicarbonate buffer pH 9.6 over night at 4°C. After washing with PBS containing 0.05% Tween-20 (PBS-T) wells were blocked with 5% skimmed milk powder in PBS-T (blocking buffer) for one hour at room temperature (RT). Following another washing step sera diluted in blocking buffer were added to wells for one hour at RT. After washing the wells were incubated with HRP-conjugated anti-mouse IgG1 or IgG2a (BD Biosciences, Heidelberg, Germany) diluted 1:1000 in blocking buffer for 1 h at RT. Subsequent to extensive washing bound antibodies were detected with chemoluminescence substrate (prepared as previously described) in a microplate luminometer with Simplicity software (ORION-96, Berthold, Bad Wildbad, Germany) and expressed as log10 transformed relative light unit (RLU) values.

### Determination of cellular immune responses

To determine the HIV Gag specific CD4+ T-cell responses, spleens were harvested 14 d after DNA priming and single cell suspension of splenocytes were prepared. After red blood cell lysis with ACK buffer, 1 × 10^6^ cells/well were plated in 96-well round-bottom plates (Nunc, Denmark). The cells were stimulated with 5 μg/ml of the HIV Gag-specific peptides (PVGEIYKRWIILGLN and SPEVIPMFSALSEGA) and 2 μg/ml anti-mouse CD3 antibody (BD Biosciences, Heidelberg, Germany) in the presence of 2 μM Monensin for 6 h at 37°C in a humidified 5% CO_2_ atmosphere. Prior to fixation with 2% paraformaldehyde cells were stained with anti mouse CD4 PerCP-eFluor® 710 and Fixable Viability Dye eFluor® 780 (eBioscience, San Diego, CA, USA). After permeabilization with 0.5% saponin in PBS/BSA/azide buffer intracellular cytokines were detected with anti-mouse TNFα Alexa Fluor® 488, anti-mouse IFNγ PE and anti-mouse IL-2 APC (BD Bioscience, Heidelberg, Germany). Following extensive washing cells were measured on a FACS Canto II (BD Bioscience, Heidelberg, Germany) and analyzed using FlowJo software (Tree Star, Inc., Ashland, OR, USA).

### In vitro co-culture experiments

Naïve untouched CD5^-^ B2 cells were isolated from single cell suspension of splenocytes and lymph node cells of SW-HEL mice with the B-Cell Isolation Kit (Miltenyi Biotec GmbH, Germany) according to the manufacturer’s instruction. Untouched CD4^+^ T-cells were isolated from single cell suspension of splenocytes and LN cells of naïve and Ad-Hgpsyn immunized BL6 mice with the T-Cell Isolation Kit (Miltenyi Biotec GmbH). CD4+ T cells were isolated from immunized mice three to four months after the last adenoviral vector immunization. DC were enriched by positive selection form spleen cell suspensions of BL6 mice with anti-CD11c magnetic beads (Miltenyi Biotec GmbH, Germany). The resulting cells were routinely >98% pure. Cells were plated in U-bottom 96-well plates at a density of 1-3 × 10^5^ CD4-T cells/well, the B-cells were added in a ratio of 2:1 (B:T) and DC in a ratio 1:5 (DC:T) and incubated for one or six days in the presence of HEL- or control VLPs in a final concentration of 100 ng of HIV-Gag per well. For indirect B-cell activation experiments, DC were plated in flat-bottom 96-well plates at the density of 1 × 10^5^ cells/well and incubated with both HEL- and control VLP for 2 h at 37°C. After intensive washing with pre-warmed medium the cells were transferred into U-bottom 96-well plate and co-cultivated with B- and T-cells as described above.

### Adoptive transfer

Splenocytes were isolated from immunized BL6 mice and pooled according to groups. CD4+ and CD8+ T-cells for adoptive transfer were purified manually from splenocyte preparations through magnetic columns using the mouse CD4+ and CD8+ T-cell isolation kits (Miltenyi Biotec, Germany; Cat.Number 130-090-860 and 130-090-859) according to the manufacturer’s manual. The purity of isolated CD4+ or CD8+ T-cells was confirmed by flow cytometry. Splenocytes and purified T cells were adoptively transferred to recipient mice by intravenous tail vein injection in a total volume of 300 μl. Each recipient mouse received the amounts of splenocytes, CD4+ T cells, or CD8+ T cells recovered from one donor mouse. For the B cell transfer, 5×10^6^ B cells purified as described for the in vitro co-culture experiments from SW-HEL mice were injected into the tail vein of the recipient BL6 mice.

### Statistical analyses

Two-sided Spearman rank correlation, two-tailed Mann–Whitney tests, and two-sided unpaired t tests were performed using the GraphPad Prism software. P values of <0.05 were considered significant.

## Competing interests

The authors declare that they have no competing interests.

## Authors’ contributions

GN planned and carried out the SIV/SHIV VLP, MSgB the HIV VLP immunization studies in mice; MT participated in the design and coordination of the study; OG and VT designed and performed the in vitro co-culture experiments; DHB acquired and evaluated the non-human primate data, KÜ conceived the study, participated in its design and coordination. All authors helped to draft the manuscript and approved the final version.
