# Correction: GagPol-specific CD4^+^ T-cells increase the antibody response to Env by intrastructural help

**DOI:** 10.1186/1742-4690-10-151

**Published:** 2013-12-10

**Authors:** Ghulam Nabi, Michael Storcksdieck Genannt Bonsmann, Matthias Tenbusch, Oliver Gardt, Dan H Barouch, Vladimir Temchura, Klaus Überla

**Affiliations:** 1 Department of Molecular and Medical Virology, Ruhr-University Bochum, 44780 Bochum, Germany; 2 Center for Virology and Vaccine Research, Beth Israel Deaconess Medical Center, Bochum, Germany; 3 Ragon Institute of MGH, MIT and Harvard, Boston, Massachusetts, USA

## Correction

After the publication of our article [1], we noted that the data points for experiment 2 in the lower panel of Figure 1 were incorrect. Statistical analyses and conclusions were not affected by this oversight. We now provide a corrected version of Figure 1 and wish to apologize for any inconvenience our error may have caused.

**Figure 1:**
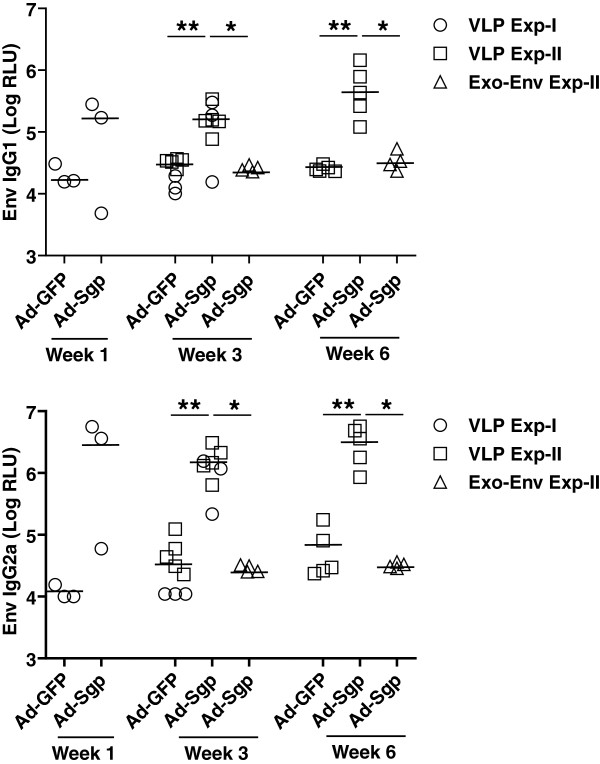
**Corrected version of figure 2 from original article **[1]**.** IgG1 and IgG2a antibody levels to SIVgp130 at 1, 3 and 6 weeks after SIV VLP boost in mice primed 6 weeks earlier with adenoviral vectors encoding SIV GagPol or GFP. Single and mean values of 3 to 9 animals per group from two independent experiments are given. *P < 0.05, **P < 0.01, Mann Whitney test.
